# Mild traumatic brain injury is associated with effect of inflammation on structural changes of default mode network in those developing chronic pain

**DOI:** 10.1186/s10194-020-01201-7

**Published:** 2020-11-23

**Authors:** Xuan Niu, Lijun Bai, Yingxiang Sun, Yuan Wang, Guanghui Bai, Bo Yin, Shan Wang, Shuoqiu Gan, Xiaoyan Jia, Hongjuan Liu

**Affiliations:** 1 Department of Critical Care Medicine, The First Affiliated Hospital of Xi’an Jiaotong University, Xi’an, China; 2 Department of Medical Imaging, The First Affiliated Hospital of Xi’an Jiaotong University, Xi’an, China; 3 Department of Radiology, Washington University School of Medicine, St. Louis, MO 63110 USA; 4 The Key Laboratory of Biomedical Information Engineering, Ministry of Education, Department of Biomedical Engineering, School of Life Science and Technology, Xi’an Jiaotong University, Xi’an, 710049 China; 5 Department of Radiology, The Second Affiliated Hospital and Yuying Children’s Hospital of Wenzhou Medical University, Wenzhou, China; 6 Department of Neurosurgery, The Second Affiliated Hospital and Yuying Children’s Hospital of Wenzhou Medical University, Wenzhou, China

**Keywords:** Mild traumatic brain injury, Posttraumatic headache, Voxel-based morphometry, Inflammation effect

## Abstract

**Background:**

Mild traumatic brain injury (mTBI) has a higher prevalence (more than 50%) of developing chronic posttraumatic headache (CPTH) compared with moderate or severe TBI. However, the underlying neural mechanism for CPTH remains unclear. This study aimed to investigate the inflammation level and cortical volume changes in patients with acute PTH (APTH) and further examine their potential in identifying patients who finally developed CPTH at follow-up.

**Methods:**

Seventy-seven mTBI patients initially underwent neuropsychological measurements, 9-plex panel of serum cytokines and MRI scans within 7 days post-injury (T-1) and 54 (70.1%) of patients completed the same protocol at a 3-month follow-up (T-2). Forty-two matched healthy controls completed the same protocol at T-1 once.

**Results:**

At baseline, mTBI patients with APTH presented significantly increased GM volume mainly in the right dorsal anterior cingulate cortex (dACC) and dorsal posterior cingulate cortex (dPCC), of which the dPCC volume can predict much worse impact of headache on patients’ lives by HIT-6 (β = 0.389, *P* = 0.007) in acute stage. Serum levels of C-C motif chemokine ligand 2 (CCL2) were also elevated in these patients, and its effect on the impact of headache on quality of life was partially mediated by the dPCC volume (mean [SE] indirect effect, 0.088 [0.0462], 95% CI, 0.01–0.164). Longitudinal analysis showed that the dACC and dPCC volumes as well as CCL2 levels had persistently increased in patients developing CPTH 3 months postinjury.

**Conclusion:**

The findings suggested that structural remodelling of DMN brain regions were involved in the progression from acute to chronic PTH following mTBI, which also mediated the effect of inflammation processes on pain modulation.

**Trial registration:**

ClinicalTrial.gov ID: NCT02868684; registered 16 August 2016.

**Supplementary Information:**

The online version contains supplementary material available at 10.1186/s10194-020-01201-7.

## Introduction

Traumatic brain injury (TBI) is a major global public health problem that affects 50 million people each year, and it is estimated that about half the world’s population will have one or more TBIs over their lifetime [1, 2]. The US Centers for Disease Control and Prevention reports that mild TBI is experienced in 70%–90% of TBI-related emergency department (ED) visits [3]. Post posttraumatic headache (PTH) is a high prevalence of disabling trauma- and pain-related disorder [4, 5], and may developed into the chronic pain in patients with TBI [1, 2]. Unexpectedly, mild TBI (mTBI) is identified as one of the most vulnerable risk to develop chronic PTH (CPTH) (prevalence rate: 72.7%–77.9%), compared with much server cases (moderate or severe TBI, prevalence rate: 29.3%–34.9%) [6]. Our recent study found that acute PTH (APTH) following mTBI led to the disrupted functional connectivity between the periaqueductal grey (PAG) and default mode network (DMN). This pain-related cognitive dysregulation may partially due to the over-attention on brain injury-related symptom. However, the underlying neurobiological basis and modulatory component remained unclear.

TBI can induce a multitude of inflammatory biomarkers perpetuating the secondary injury to the brain [7, 8], which upregulates central nerve system (CNS) excitability contributing to the generation and persistent of concomitant headache [9–11]. Multiple pain disorders have altered grey matter volume (GMV) within the pain matrix [12, 13]. Notably, peripheral inflammatory cytokines/chemokines can interact with multiple central pathways as a principal channel for inflammation-brain communication in the development of pain states [14, 15]. Considering the potential effect of trauma-induced systemic inflammation on brain cell reaction [16, 17], we hypothesized that the inflammatory cytokines level might lead to changes in pain perception via inflammation-brain mechanism [18, 19], specifically by affecting cortical morphology alternations in pain-related cognitive modulation following mTBI [20].

The present study investigated modifications of GM volume to identify those brain regions related to the emergence and persistence of pain condition known as PTH following mTBI, and contrasted patients without PTH, in addition to healthy controls. This study was aimed to examine whether GMV changes mediated the relationship between inflammation and PTH in acute mTBI patients. Longitudinally, we hypothesized that both inflammatory response and brain morphological alterations contributed to those mTBI patients who finally developed into CPTH.

## Material and methods

### Participants

Seventy-seven patients (45 male, ages of 34.7 ± 12.2 years, education level of 8.6 ± 3.8 years) with mTBI and forty-two matched healthy controls (HC, 21 male, ages of 35.3 ± 11.2 years, education level of 10.5 ± 5.2 years) were recruited in the study (Clinical trial: NCT02868684). All consecutive patients from the local emergency department (ED) with non-contrast head CT due to acute head trauma enrolled as the initial population. Inclusion criteria for mild TBI were based on the World Health Organization’s Collaborating Centre for Neurotrauma Task Force [21]. Mild TBI patients were excluded:1) history of a previous brain injury, preexisting headache, neurological disease, long-standing psychiatric condition, or concurrent substance or alcohol abuse, 2) structural abnormality on conventional neuroimaging (CT and MRI), 3) intubation and/or presence of a skull fracture and administration of sedatives, 4) the manifestation of mild TBI due to medications by other injuries (e.g., systemic injuries, facial injuries, or spinal cord injury), 5) other problems (e.g., psychological trauma, language barrier, or coexisting medical conditions), 6) caused by penetrating craniocerebral injury. Patients with structural abnormality on conventional neuroimaging and a premorbid condition, such as history of a previous brain injury, preexisting headache, neurological disease, concurrent substance or alcohol abuse were excluded. MRI scanning for mTBI patients was originally evaluated within 7 days post-injury (acute phase) and follow-up at 3 month post-injury (chronic phase). Measures for patients were circulating markers of inflammation, clinical and neuropsychological assessments within 48 h of MRI scans.

MTBI patients were divided into two groups based on the presence of PTH at the acute stage (within 7 days post-injury): mTBI with and without acute posttraumatic headache (APTH) according to the Third Edition of the International Classification of Headache Disorders (ICH-D-3) [22]. If the headache persists for longer than 3 months after head trauma, it is described as the chronic/persistent PTH (CPTH/PPTH).

Healthy subjects carefully screened for history of acute/chronic pain, neurological or psychiatric disorder, were also recruited. Forty-two age-, sex- and education-matched healthy volunteers completed an identical neuroimaging scan and assessments at a single time point as a control group. The study was approved by the local ethics committee in accordance with the Declaration of Helsinki. Written informed consent was obtained from all participants. (see online supplementary eMethods).

### Serum biomarker collection and assay

Serum samples for both patients and controls were collected in the morning around 7–8 am. Sample were aliquoted and stored at − 80 °C until the time of assay after collection and centrifugation. Serum cytokine levels (pg/mL) were measured using reagents on a Luminex multiplex bead system (Luminex Austin, TX, USA). A fluorescence detection laser optic system was used to analyze binding of each individual protein on the microsphere simultaneously, which permits multiplexed analysis of several analytes in one sample. Intra- and inter-assay coefficients of variation observed for Luminex quantification were less than 20% and 25%, respectively. Samples with levels that were undetectable by the assay were set to the value of 0.01 pg/mL. The criteria for cytokines selection were mainly based on whether it’s related to TBI or clinical symptoms such as PCS and pain function [23, 24]. The cytokines included (i) the archetypal pro-inflammatory cytokines: IL-1β, IL-6 and IL-12, and the anti-inflammatory cytokines IL-4, IL-10; (ii) chemokine (C-C motif) ligand 2 or monocyte chemoattractant protein-1(CCL2 or MCP-1) and member of the CXC chemokine family (CXCL8) IL-8; (iii) interferon-γ (IFN-γ); and (iv) tumor necrosis factor α (TNF-α).

### Clinical evaluations and pain symptom measurement

Clinical evaluations included post-injury days, duration for both the posttraumatic amnesia (PTA) and loss of consciousness (LOC). Intensity of pain symptoms were assessed by the Visual Analogue Scale (VAS, range 0–10) [25]. The Pain VAS confines to patient-report (PR) measures including pain subscales for the current headache and current general pain as well as mean, best and worst levels of general pain intensity experienced in the preceding week. The VAS ranges from 0 to 10, with 0 meaning “no pain at all” and 10 “the worst possible pain” and reliably tested in previous reports [26, 27]. The impact of headache on patients’ lives was also evaluated with the Short-Form Headache Impact Test (HIT-6) [28, 29].

### Neuropsychological assessment

A comprehensive neuropsychological assessments included: *i)* Trail-Making Test Part A and Digit Symbol coding score from the Wechsler Adult Intelligence Scale III (WAIS-III) to examine cognitive information processing speed; *ii)* Forward Digit Span and Backward Digit Span from the WAIS-III to assess immediate auditory span, working memory, and executive function; *iii)* Verbal Fluency Test to assess verbal fluency including language ability, semantic memory and executive function; *iv)* Depression severity was assessed using the Beck Depression Inventory (BDI-II); *v)* Posttraumatic stress disorder (PTSD) Checklist - Civilian Version (PCL-C); *vi)* Fatigue Severity Scale, Insomnia Severity Index. In addition, post concussive symptoms (PCS) were measured with the Rivermead Post-Concussion Symptom Questionnaire (RPQ) [30] consisting of 16 items, which was specifically developed to assess the severity of symptoms experienced after brain injury.

### Image acquisition

The protocol for scanning included a non-contrast CT scan for acute head injury. MRI scanning was conducted on 3 T MRI scanner (GE 750) and included the T1-weighted 3D BRAVO sequence, conventional T1- and T2-weighted image, and susceptibility weighted imaging (SWI) (see online supplementary eMethods).

### MRI data processing

The T1-MRI images were processed using the Computational Anatomy Toolbox (CAT12) in Statistical Parametric Mapping 12 (SPM12; https://www.fil.ion.ucl.ac.uk/spm/software/spm12/). Anatomical images were firstly segmented into the gray matter, white matter, and cerebrospinal fluid (CSF), spatially normalized into the Montreal Neurological institute (MNI) template space, and then smoothed with an isotropic Gaussian kernel of 8 mm full width at half maximum. Group differences on GMV using the total intracranial volume, the white matter volume, age, and sex as covariants were performed with a cluster forming (voxel-wise) threshold of uncorrected *p* < 0.001 and then corrected for multiple comparisons at the cluster level (*p* < 0.05, family-wise error (FWE) rate correction). To explore the influence of depression on GMV, we repeated the above analyses of between-group differences after adding the BDI-II score as covariate. Absolute threshold masking was set at 0.1 to avoid edge effects between gray and white matter. Based on the previous studies of pain-related diseases [31], we performed region of interest (ROI) analysis within the pain matrix including the anterior and posterior cingulate cortex, prefrontal cortex, insula, hippocampus, middle and inferior temporal gyrus, and thalamus, according to the Brodmann template using Slice Viewer in REST V1.8.

### Mediation analysis

To examine whether regional GMV could mediate the effect of inflammation cytokines on pain symptom in the acute PTH, a mediation analysis was performed by using the PROCESS tool [32] as implemented in SPSS v.21. Firstly, a stepwise regression model was used to calculate the odd ratio of the serum biomarker for pain symptom measurements in mTBI patients. Secondly, based on the results of the stepwise regression analysis, resulting serum biomarker was entered as the independent variable, pain symptom measurements as the dependent variable. The mean GM volume for each region showing significant group difference was tested separately as the mediator variable in the mediation analyses, and age, sex, education and injury time as covariates of no interest .

### Longitudinal analysis in CPTH

Changes of the acute serum biomarker related to the pain symptom were compared over time from acute to 3 months post-injury within each group (mTBI + CPTH and mTBI – CPTH groups), using the general linear model of repeated measure analysis of variance (RM-ANOVA) respectively.

### Statistical analysis

Statistical analysis was performed using the Statistical Package SPSS version 20. The Shapiro-Wilk W test was used to test for normality distribution of all continuous variables. Logarithmic transformations were computed if those variables that were not normally distributed (ie, the inflammatory biomarkers). The independent two-sample t-test and the Mann-Whitney test were used to compare group differences based on data normality. Chi-square analyses were applied to assess categorical variables. Continuous variables were compared between three groups using one-way analysis of variance (ANOVA), Bonferroni’s post hoc test and Kruskal-Wallis H Tests. *P* < 0.05 were considered to indicate a significant difference. Between-group difference in both acute and chronic post-injury GM volume abnormalities was conducted using the general linear model in the SPM12. Using a conjunction analysis [33], the different subgroups of mTBI patients (with PTH and without PTH) were compared to each other and to controls in both acute and chronic phases respectively. Additionally, the relationship between the GM volume for each identified ROI and pain measures were conducted using the multivariate linear regression analysis after adjusting for confounding covariates (age, sex, education and injury time). For each model, pain symptom measurements (P-VAS and HIT-6) were entered as dependent variables and brain regions showing significant GMV group difference were entered as independent variables. This procedure was repeated for both initial and follow-up data, and corrected for multiple comparisons using Bonferroni correction.

## Results

### Demographic, clinical and neuropsychological measures

Demographic, behavioral and conventional MRI characteristics of the mTBI subgroups (for both initial and follow-up stages) and HC participants were summarized in the Table 1. In the acute phase (2.39 ± 1.47 days, range: 0–6 days), a seventy-seven patients were divided into two subgroups: 1) mTBI + APTH, 60 individuals with APTH after mTBI, 2) mTBI – APTH, 17 individuals with mTBI without headache. Forty-two matched healthy controls were recruited in the study. Fifty-four patients (70.1%) from the original sample (mTBI + CPTH: 15; mTBI – CPTH: 39) returned for their follow-up visit at 3 months post-injury (111.4 ± 22 days, range: 93–198). At follow-up, there were significant difference between groups in years of age and education. Post-hoc analysis further showed mTBI + CPTH was significantly older than that of mTBI – CPTH (*p* = 0.007) and HCs (*p* = 0.046). The years of education in the mTBI + CPTH was lower than that of HCs (*p* = 0.017). Twenty-three patients were excluded for refusing or indefinitely postponing follow-up request. 25% of mTBI + APTH patients converted to persistent PTH known as CPTH during a follow-up 3 month post-injury.

**Table 1:** Demographic and clinical characteristic for acute/chronic mTBI subgroups and HC participants

	HCs ***n*** = 42	Acute phase		***P*** value*	Chronic phase		***P*** value^**+**^
		mTBI + APTH ***n*** = 60	mTBI - APTH ***n*** = 17		mTBI + CPTH ***n*** = 15	mTBI - CPTH ***n*** = 39	
**Demographic**							
Age in years	35.3 ± 11.2	37.47 ± 12.6	30.4 ± 12.8	0.109	44.1 ± 12.8	32.8 ± 12.2	0.009^e^
Males (%)^C^	21 (50%)	37 (61.7%)	8 (47%)	0.381	6 (40%)	24(61.6)	0.313
Years of education	10.5 ± 5.2	8.6 ± 4.0	9.0 ± 2.8	0.095	7.0 ± 4.8	9.2 ± 3.5	0.016^f^
**Pain measurement**							
Present headache pain intensity^K^	0	4(1–10)	0	*p* < .0001^a^	2(1–4)	0	*p* < .0001^g^
HIT-6^K^	36	48(36–68)	36	*p* < .0001^a^	50(36–68)	36	*p* < .0001^g^
Present general pain intensity^K^	0	1(0–6)	0(0–4)	*p* < .0001^a^	0(0–2)	0(0-1)	*p* < .0001^g^
Average pain intensity	0	4(1–8)	0(0–4)	*p* < .0001^a^	2(0–5)	0(0-4)	*p* < .0001^g^
over the past week^K^							
Best pain intensity^K^	0	0(0–8)	0(0–2)	*p* < .0001^a^	0(0–2)	0	*p* < .0001^g^
Worst pain intensity^K^	0	6(2–10)	0(0–6)	*p* < .0001^a^	4(0–6)	0(0-6)	*p* < .0001^g^
**Neuropsychological ratings**							
PCL-C^K^	28^A^ /23.5^B^	78.0	76.3	*p* < .0001^b^	81.7	62.6	*p* < .0001^h^
WAIS-III Coding	36.1 ± 15.8	33.7 ± 16.0	31.3 ± 13.8	*p* < .0001^c^	33.7	50.8	*p* = .126
Trail Making A (s)	46.7 ± 16.8	61.1 ± 49	43.4 ± 32.0	0.118	68.7 ± 59.4	38.6 ± 22.3	*p* = .021^i^
Forward Digit Span	45.9 ± 33.1	10.2 ± 9.4	8.5 ± 2.8	0.358	7.7 ± 1.7	8.7 ± 1.5	*p* = .137
Backward Digit Span	8.3 ± 1.5	4.2 ± 2.1	4.2 ± 1.7	0.693	4.1 ± 1.9	4.6 ± 1.5	*p* = .623
Verbal Fluency Test	4.5 ± 2	14.8 ± 5.9	15.0 ± 4.8	0.003^b^	17.3 ± 7.0	17.8 ± 5.4	*p* = .668
BDI-II	18.7 ± 6.4	5.2 ± 5.1	4.2 ± 4.5	*p* < .0001^c^	5.8 ± 3.9	4.4 ± 2.5	*p* < .0005^j^
FSS^K^	60.5^A^/48.5^B^	57.7	67.1	0.138	48.5	48.5	*p* = 1
ISI	1.6 ± 2.3	7.3 ± 5.8	4.3 ± 3.7	*p* < .001^d^	4.4 ± 4.3	2.9 ± 3.3	*p* = .008^k^
**MRI characteristics**							
Total intracranial volume	1467.2 ± 149.4	1462.8 ± 141.4	1446.2 ± 119.1	0.873	1454.3 ± 153.9	1448.5 ± 115.1	0.825
Gray matter volume	631.7 ± 70.5	627.0 ± 64.9	602.4 ± 48.5	0.283	617.9 ± 49.9	627.0 ± 64.0	0.779
White matter volume	519.9 ± 66.9	508.5 ± 52.1	480.4 ± 57.8	0.067	505.9 ± 59.7	493.9 ± 51.1	0.151
Cerebrospinal fluid	314.8 ± 89.7	326.4 ± 106.2	374.5 ± 119.2	0.129	329.7 ± 94.3	327.0 ± 99.1	0.799

Mean ± standard deviation are reported. Sex is reported as frequencies. ^C^ Chi-square; ^K^ Kruskall Wallis (median (range) reported for pain measurement; mean ranks reported for the other measurement); ^A^ Comparisons of mTBI + APTH, mTBI–APTH and HCs; ^B^ Comparisons of mTBI + CPTH, mTBI–CPTH and HCs

*Abbreviations*: *HIT-6* Short Form Headache Impact Test, *PCL-C* PTSD Checklist – Civilian Version, *WAIS* Wechsler Adult Intelligence Scale – Third Edition, *BDI-II* the Beck Depression Inventory- Second Edition, *FSS* Fatigue Severity Scale, *ISI* Insomnia Severity Index, *mTBI + APTH* mild traumatic brain injury and acute post-traumatic headache, *mTBI–APTH* mild traumatic brain injury without acute post-traumatic headache, *HCs* healthy controls, *mTBI + CPTH* mild traumatic brain injury and chronic post-traumatic headache, *mTBI–CPTH* mild traumatic brain injury without chronic post-traumatic headache

* For comparisons among acute mTBI subgroups and HC participants. ^+^ For comparisons among chronic mTBI subgroups and HC participants

^a^mTBI + APTH > mTBI – APTH, HCs; *p* < .0001

^b^mTBI + APTH, mTBI – APTH > HCs; *p* < .005

^c^mTBI + APTH, mTBI – APTH < HCs; *p* < .005

^d^mTBI + APTH > HCs; *p* < .001

^e^mTBI + CPTH > mTBI – CPTH, HCs; *p* < .05

^f^mTBI + CPTH < HCs; *p* < .05

^g^mTBI + CPTH > mTBI – CPTH, HCs; *p* < .0001

^h^mTBI + CPTH, mTBI – CPTH > HCs; *p* < .0001

^i^mTBI + CPTH > mTBI – CPTH; *p* < .05

^j^mTBI + CPTH, mTBI – CPTH < HCs; *p* < .00005

^k^mTBI + CPTH > HCs; *p* < .05

In both acute and chronic stages, there were significant differences among the three groups for pain ratings and impact of pain on patients’ lives, such as 5-item subscale of P-VAS scores and HIT-6 using Kruskal-Wallis H Tests (*p* < 0.0001). Post-hoc analysis further showed mTBI + APTH presented more pain complaints and higher HIT-6 scores, compared with both mTBI – APTH and HCs. Similarly, mTBI + CPTH also experienced more pain complaints and higher HIT-6 scores than that of the mTBI – CPTH and HCs. Additionally, mTBI + APTH group reported more PCS complaints than that of the mTBI – APTH group (*p* = 0.007). Additionally, in comparison with the mTBI – CPTH group, mTBI + CPTH group developed more PCS complaints (*p* = 0.003). Other key clinical measurement of TBI severity showed no between-group differences in patients (Table 2).

**Table 2:** Patients’s clinical data

**Initial (acute phase)**	mTBI + APTH	mTBI - APTH	P (2-tailed)
**Duration after onset of mTBI, day**	2.42 ± 1.45	2.29 ± 1.57	0.65
**Duration of LOC (min)**	9.70 ± 8.94	10.76 ± 12.96	0.78
**Duration of PTA (h)**	0.28 ± 1.01	0.18 ± 0.53	0.86
**PCS (RPQ-6)**	10.37 ± 6.95	5.59 ± 3.18	0.007
**Follow-up (chronic phase)**	mTBI + CPTH	mTBI - CPTH	P (2-tailed)
**Duration after onset of mTBI, day**	104 ± 12	114.1 ± 23.92	0.14
**Duration of LOC (min)**	7.9 ± 6.2	9.74 ± 10.2	0.433
**Duration of PTA (h)**	0.2 ± 0.56	0.15 ± 0.49	0.767
**PCS (RPQ-6)**	15 ± 7.09	8.13 ± 5.48	0.003

Mean ± standard deviation are reported. *mTBI + APTH* mild traumatic brain injury and acute post-traumatic headache, *mTBI – APTH* mild traumatic brain injury without acute post-traumatic headache, *mTBI + CPTH* mild traumatic brain injury and chronic post-traumatic headache, *mTBI –CPTH* mild traumatic brain injury without chronic post-traumatic headache, *PCS* Post concussive symptoms, *RPQ* Rivermead Post-Concussion Symptoms Questionnaire, *LOC* Loss of consciousness, *PTA* Posttraumatic amnesia

### Global tissue volume changes

For both initial and follow-up stages, there were no significant differences in the total intracranial volume (*p* = 0.873; *p* = 0.825), gray matter volume (*p* = 0.283; *p* = 0.779), white matter volume (*p* = 0.067; *p* = 0.151), or cerebrospinal fluid volume (*p* = 0.129; *p* = 0.799) among these three groups (Table 1).

### Regional GM volume abnormalities at acute post-injury stage - effect of onset of PTH

**Fig. 1:**
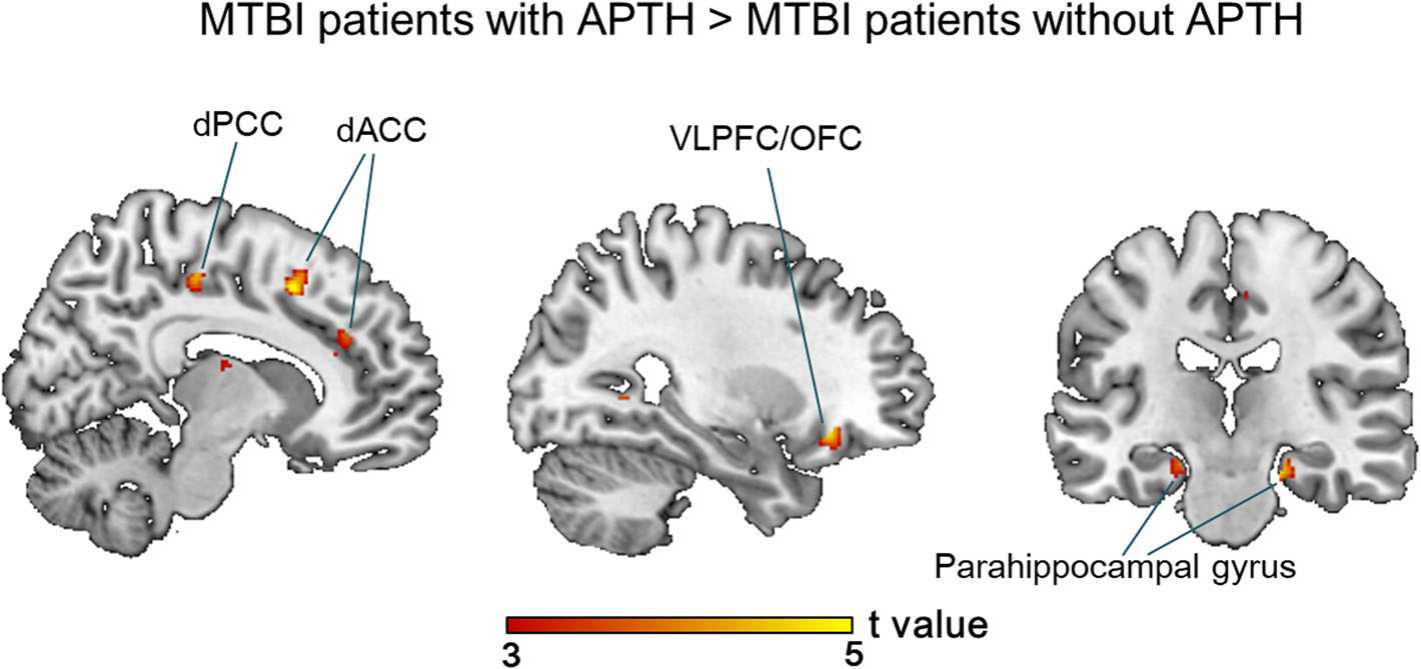
GM volume changes in mTBI + APTH. Areas showing GM volume changes in patients with mTBI + APTH, compared with HC and mTBI – APTH groups (conjunction *p* < 0.05, FWE corrected), represented on a high-resolution T1-weighited template. Regions of increased GM volume are represented in red (color-coded for their *t* value). dACC = dorsal anterior cingulate cortex, dPCC = dorsal posterior cingulate cortex, VLPFC = ventrolateral prefrontal cortex, OFC = orbitofrontal cortex. mTBI + APTH = mild traumatic brain injury and acute post-traumatic headache, mTBI – APTH = mild traumatic brain injury without acute post-traumatic headache, HCs = healthy controls

For the ROI-based analysis, mTBI + APTH exhibited increased GM volume in the right ventrolateral prefrontal cortex/orbitofrontal cortex (VLPFC/OFC), dorsal anterior cingulate cortex (dACC), dorsal posterior cingulate cortex (dPCC), and bilateral parahippocampal gyrus compared with both mTBI – APTH and HC groups (Table 3 and Fig. 1). There was no significantly decreased GM volume of regions in the mTBI + APTH groups. No other regions that fell outside a priori regions of interest was detected.

**Table 3:** Regions showing significant GM volume changes at initial and follow-up among different subgroups of mTBI patients and HCs

Comparisons	Findings	Cerebral regions	Area (Brodmann area)	Cluster size	t	Peak coordinate		
				Voxels		x	y	z
Initial GM volume abnormalities (acute phase)								
**mTBI + APTH** **vs** **Control groups**^**a**^	Increased GM volume	L, R Parahippocampal gyrus	Parahippocampal (BA 35)	54	4.38	20	−20	−2
		R Anterior cingulate	dACC (BA 32)	105	4.68	11	15	0
		R Posterior cingulate	dPCC (BA 31)	47	4.07	6	−24	45
		R Inferior frontal gyrus	VLPFC/OFC (BA 47, 10)	39	4.50	26	27	48
Follow-up GM volume abnormalities (chronic phase)								
**mTBI + CPTH vs Control groups**^**b**^	Increased GM volume	L Anterior cingulate	dACC (BA 32)	64	4.54	− 11	12	41
		L Posterior cingulate	dPCC (BA 31)	78	4.47	−11	− 29	36
		L Precentral gyrus	M1 (BA 4)	53	4.20	−18	−32	69
		L Temporal gyrus	ITG (BA 20)	66	4.67	−59	−48	−15
		L Cerebellar declive	–	65	5.14	−45	−59	−27
**mTBI + CPTH vs Control groups**^**c**^	Decreased GM volume	R Inferior frontal gyrus	DLPFC/OFC (BA9,10)	53	4.92	9	59	33

Data were thresholded of *P* < 0.05 with FWE correction at the cluster level for multiple comparison. Peak coordinates refer to the MNI atlas

*dACC* dorsal anterior cingulate cortex, *dPCC* dorsal posterior cingulate cortex, *VLPFC* Ventrolateral prefrontal cortex, *OFC* Orbitofrontal cortex. *M1* primary motor cortex, *ITG* Inferior temporal gyrus, *DLPFC* Dorsolateral prefrontal cortex, *mTBI + APTH* Mild traumatic brain injury and acute post-traumatic headache, *mTBI – APTH* Mild traumatic brain injury without acute post-traumatic headache, *HCs* Healthy controls. *mTBI + CPTH* mild traumatic brain injury and chronic post-traumatic headache, *mTBI – CPTH* mild traumatic brain injury without chronic post-traumatic headache, *FWE* Family-wise error

^a^Conjunction analysis of mTBI + APTH vs mTBI – CPTH and mTBI + APTH vs HCs corrected for age, sex, the white matter volume and total intracranial volume (*p* < 0.05, FWE corrected)

^b^Conjunction analysis of mTBI + CPTH vs mTBI – CPTH and mTBI + CPTH vs HCs corrected for age, sex, the white matter volume and total intracranial volume (*p* < 0.05, FWE corrected)

^c^Conjunction analysis of mTBI + CPTH vs mTBI – CPTH and mTBI – CPTH vs HCs corrected for age, sex, the white matter volume and total intracranial volume (*p* < 0.05, FWE corrected)

### Regional GM volume abnormalities at chronic post-injury stage - effect of PTH progression

**Fig. 2:**
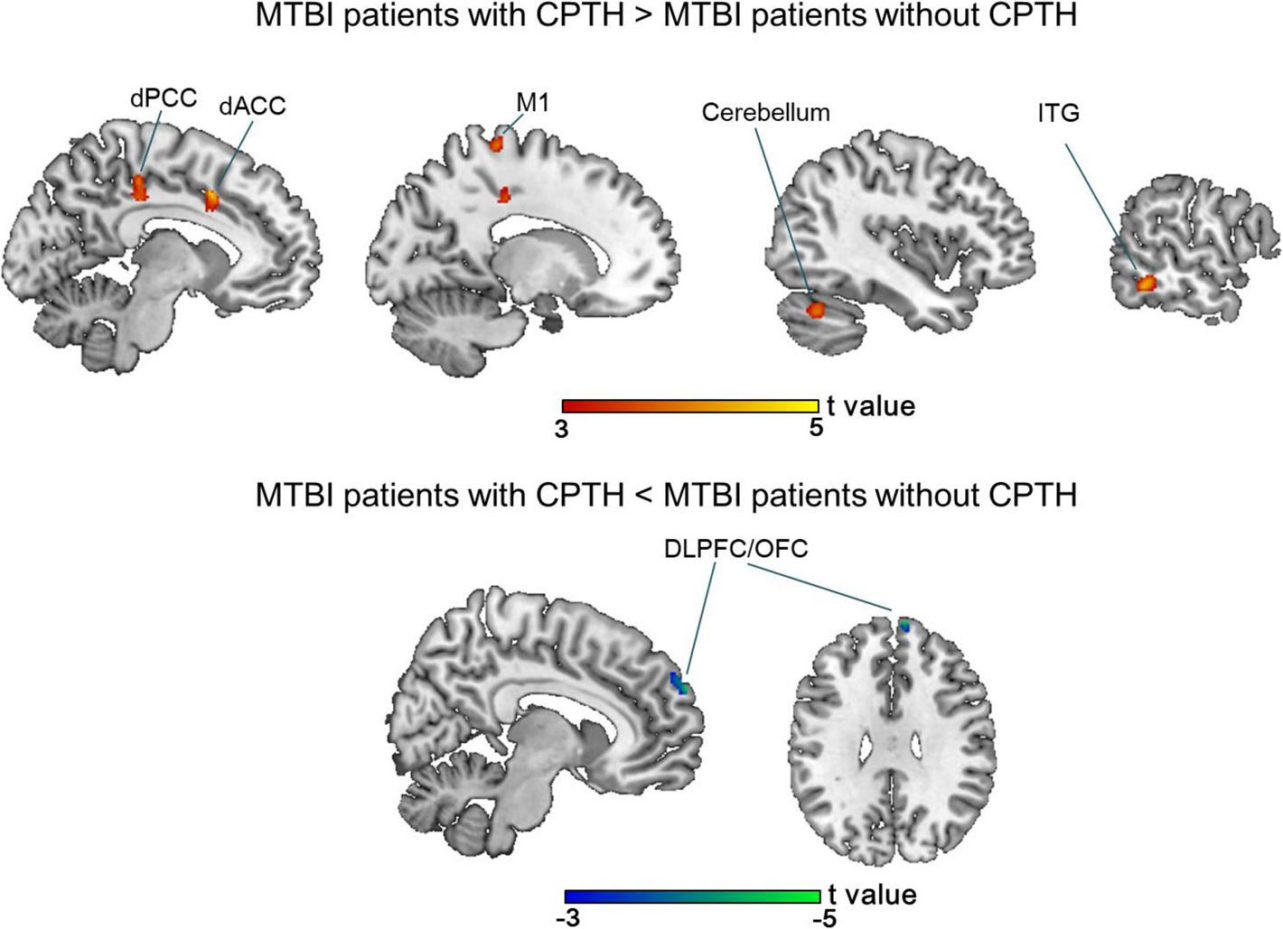
GM volume changes in mTBI + CPTH. Areas showing GM volume changes in patients with mTBI + CPTH, compared with HC and mTBI – CPTH groups (conjunction *p* < 0.05, FWE corrected), represented on a high-resolution T1-weighited template. Regions of increased GM volume are represented in red (color-coded for their t value), and regions of decreased GM volume are shown in blue (color-coded for their t values). dACC = dorsal anterior cingulate cortex, dPCC = dorsal posterior cingulate cortex, M1 = primary motor cortex, ITG = inferior temporal gyrus, DLPFC = dorsolateral prefrontal cortex, OFC = orbitofrontal cortex. mTBI + CPTH = mild traumatic brain injury and chronic post-traumatic headache, mTBI – CPTH = mild traumatic brain injury without chronic post-traumatic headache, HCs = healthy controls

The increased GM volumes in the dACC and dPCC were still persistent in the mTBI + CPTH, while there were also decreased GM volume in the right dorsolateral prefrontal cortex/orbitofrontal cortex (DLPFC/OFC), in comparison with both the mTBI – CPTH and HC groups (Table 3 and Fig. 2). In addition, mTBI + CPTH group also showed brain volume increase in the left primary motor cortex (M1) and cerebellum by using exploratory whole-brain analysis of GMV. The repeated analyses with adding the BDI-II score as covariate at both acute and chronic post-injury stage yielded similar results. Thus, the possible influence of depression level on GM volume changes can be excluded.

### Regression analysis

Further regression analysis was restricted within the clusters showing significant between-group differences in the GMV for both acute and 3-month follow-up (Table 3). During the acute stage, higher levels of HIT-6 were significantly correlated with increased GM volume in the right dACC (β = 0.333, *p* = 0.005) and dPCC (β = 0.459, *p* = 0.001) in the whole mTBI cohort, while still significant in the right dPCC (β = 0.389, *P* = 0.007) in mTBI + APTH group. There was no correlation between GMV and pain symptom measured by P-VAS and HIT-6 at follow-up stage. Further, Serum biomarker was used to build a stepwise regression model with pain symptom measurement. Only higher CCL2 level in the acute stage was associated with greater severity of HIT-6 scores in the whole mTBI cohort during the acute phase and selected for further mediation analysis (*p* = 0.002, effect size = 0.355) (Table 4). In addition, there were significant differences among the three groups for acute CCL2 level using one-way analysis of variance (ANOVA) (F (2, 116) = 3.165, *p* = 0.046). Post-hoc analysis further showed that CCL2 was significantly elevated in mTBI + APTH group, compared with mTBI–APTH group (*p* = 0.039). It indicated a nonstatistically significant trend of increased CCL2 level in mTBI + APTH group, compared with the HCs group (*p* = 0.052). There was no significant difference in CCL2 level between the mTBI–APTH and HCs group (*p* = 0.534).

**Table 4:** Stepwise multiple regression analysis

Inflammatory Level	Correlation coefficient	T	P	Partial correlation	Collinearity statistics	
					Tolerance	VIF
CCL2^a^	0.355	3.283	0.002^*^	0.355	1.000	1.000
IL-1β^b^	0.015	0.139	0.890	0.016	1.000	1.000
IL-4^b^	0.010	0.093	0.927	0.011	0.999	1.001
IL-6^b^	− 0.106	−0.981	0.330	− 0.113	0.994	1.006
IL-8^b^	− 0.027	−0.185	0.854	− 0.022	0.542	1.843
IL-10^b^	0.025	0.229	0.819	0.027	0.991	1.009
IL-12^b^	0.017	0.156	0.876	0.018	1.000	1.000
IFN-γ^b^	0.038	0.347	0.730	0.040	0.999	1.001
TNF-α^b^	0.032	0.291	0.772	0.034	1.000	1.000

^a^Entered variables

^b^Excluded variables

*p < .05

Outcome: HIT-6

### Mediation analysis

**Fig. 3:**
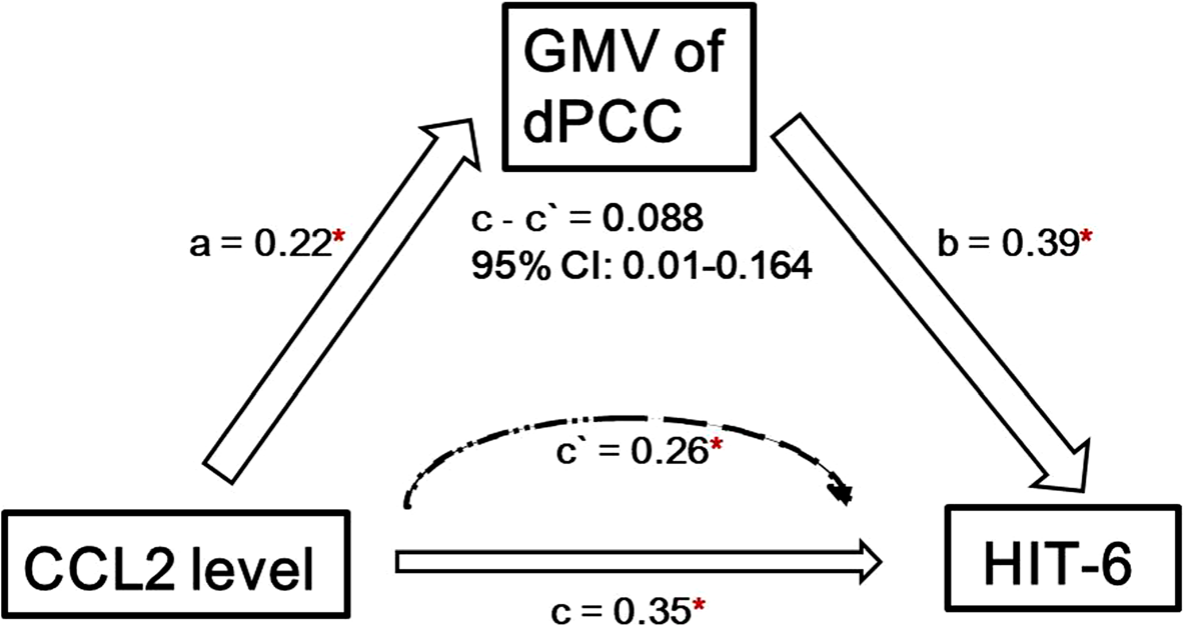
Mediation model. The relationship among CCL2 level, GMV of dPCC from conjunction analysis and HIT scores. Alteration of gray matter volume in the dPCC mediates the relationship between CCL2 level and HIT scores in early mTBI patients. Covariates (age, sex, education, injury time) were included in the model. Abbreviations: dPCC = dorsal posterior cingulate cortex; CCL2 = C-C motif chemokine ligand 2; HIT = short form headache impact test

We tested whether GM volume (i.e. increased GM regions) mediates the association between CCL2 levels and HIT-6 scores. The independent factor was CCL2 levels, and dependent variable was headache impact severity indicated by the HIT-6, with identified GM volume alterations as mediators. Results indicated that increased volume in the dPCC partially mediated CCL2 level on HIT-6 scores (Fig.3, mean [SE] indirect effect, 0.088 [0.0462], 95% CI, 0.01–0.164) in acute mTBI patients.

### Longitudinal analysis of CPTH

**Fig. 4:**
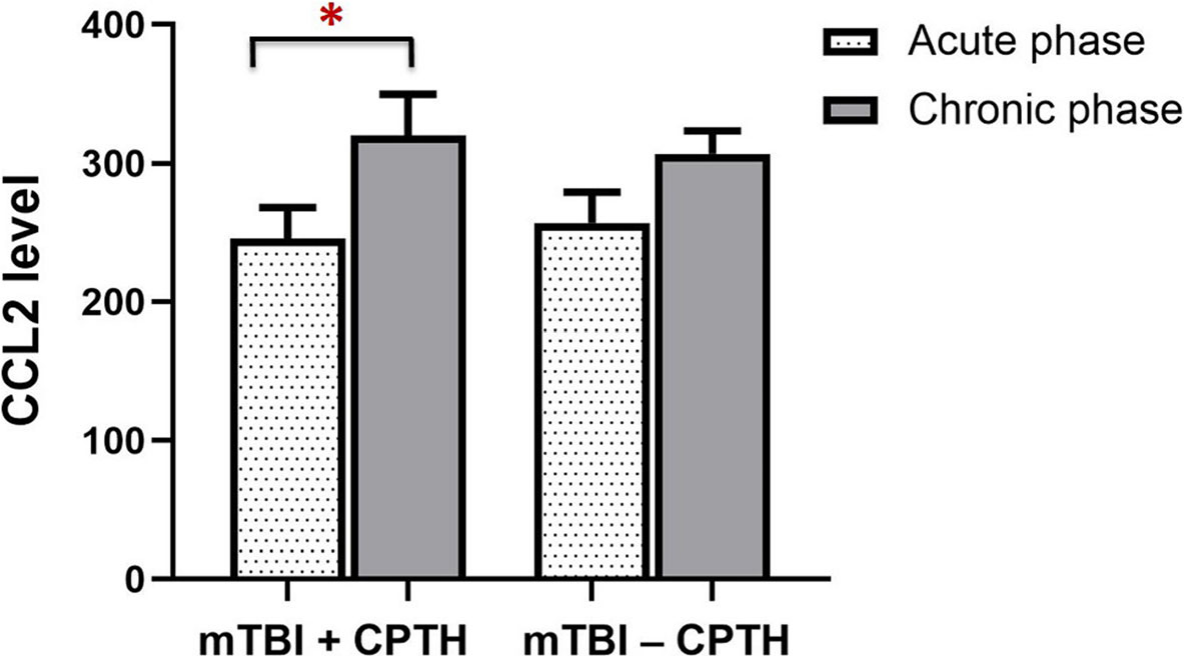
CCL2 level changes at acute and chronic phase post-injury. The CCL2 level significantly increased between the acute and chronic phase post-injury in the mTBI + CPTH group (*p < 0.05, repeated measures analysis of covariance [RM-ANCOVA]), but not in the mTBI – CPTH group (*p* = 0.08, RM-ANCOVA). mTBI + CPTH = mild traumatic brain injury and chronic post-traumatic headache, mTBI – CPTH = mild traumatic brain injury without chronic post-traumatic headache

The CCL2 level significantly increased from acute stage to 3 months post-injury only in the mTBI + CPTH group (F (1, 14) = 6.53, *p* = 0.023), but not in the mTBI – CPTH group (F (1, 38) = 3.23, *p* = 0.08) (Fig. 4). In addition, increased GMV of the dPCC and dACC in acute PTH also exhibited persistent increases in mTBI + CPTH group, compared with both mTBI – CPTH and HC groups.

## Discussion

The major findings were increased inflammation levels accompanied by increased GMV of the dPCC and dACC in mTBI patients with APTH, relative to both the control groups (mTBI – APTH and HCs). The inflammation effect on the headache impact was partially mediated by the GMV of dPCC. Longitudinally, greater upregulation in the CCL2 level accompanied by consistently increased GMV in the dPCC and dACC contributed to those patients who finally developed into the CPTH.

The observed pattern of increased GMV differs from some previous studies that commonly reported decreased GMV in pain-related disorders, partly due to the fact that APTH patients experienced a traumatic exposure that can lead to both physical and psychological reactivity, and thus developed an adaptive anti-nociceptive mechanism manifesting as regional hypertrophy in pain-related areas [34]. Also, prior structural studies in PTH were typically conducted on patients with chronic course of headache, confirming that patients with persistent PTH had significantly reduced grey matter volume (GMV) or less cortical thickness [13, 35]. To our knowledge, it remained unknown that if brain morphologic abnormality is associated with the acute onset of PTH. Our findings indicated that acute brain trauma-related headache (within 7 days) may shift the brain into a state that fostered rapid defence mechanisms seen in increased GMV. Interestingly, the increased GMV pattern was found in regions primarily located in the default mode network (DMN), including dACC, dPCC and parahippocampal gyrus. Our previous study had found disruption of periaqueductal grey-DMN functional connectivity in APTH after mTBI. Thus, these findings complement and extend existing literature by indicating a potential structural basis for the disrupted functional connectivity of the DMN involved in the antinociceptive descending modulation network following mTBI [36].

Neuropsychological assessment showed that both the mTBI + APTH and mTBI + CPTH groups reported higher insomnia severity index (ISI) scores than HC group, which support the notion that PTH has impacts on sleep quality and can lead to poor recovery after mTBI [37]. Correlation analysis further revealed that the higher GMV in the right dACC and dPCC contributed to the impact of PTH on the poorer quality of life. DMN dysfunction has been reported to be involved in both acute and chronic pain conditions, serving as a potential biomarker for pain-related cognitive regulation (i.e., attention impairment) [38, 39]. Importantly, our previous study have demonstrated that patients with APTH exhibit more attentions on injury-related pain symptoms [20]. In the current study, mTBI + CPTH group showed worse information processing speed (IPS) performance indicated by higher Trail Making A scores than mTBI – CPTH group, accompanying with the persistent GM increase in the dACC and dPCC, which can be engaged in cognitive regulation [40]. These structural changes may be coupled with alterations in the high-order brain areas within the DMN in mTBI + APTH group [41], reflecting the DMN dysfunction in pain cognitive modulation. It also suggested that morphometric alterations can be an appropriate parameter for monitoring PTH in the early stage following mTBI.

The current study also provided some promising to explore the cellular basis for such increased GMV. We found that increased CCL2 level is an independent risk for more headache impact on lives in patients with mTBI. CCL2 is one of chemokines that increases rapidly after various forms of experimental mTBI [42, 43], contributing to secondary brain damage through attracting monocytes to sites of injury and inflammation [44]. Moreover, mTBI-induced disruption of the blood-brain barrier (BBB) [45] allows the passage of inflammatory cells out of the injured brain, and initiates to an elevated systemic immune response to the genesis of nociceptor hypersensitivity post-TBI [11]. Of particular interest is the finding of macroscopic GM hypertrophy in the dPCC (a core region in the DMN), which is related to the arousal, self-reference and breadth of attention [40], can mediate the contribution of circulating inflammatory biomarker (i.e. CCL2 level) on pain impact severity during early stage after mTBI. This inflammation-brain communication can be supported by the evidence that astrocytes, which constitute 90% cortical tissue volume, can be activated by proinflammatory cytokines released in the early stage of inflammation and exhibit morphological changes (e.g. hypertrophy) [46, 47]. Thus, we speculate that GM tissue cells can become hyper-reactive by trauma-induced neuroinflammatory effect, and then undergo plastic changes reflected by GMV increases. Unfortunately, VBM-based analysis was limited to determine the specific histopathology underlying GM macrostructural changes. Further study still need to explore the neural mechanism underpinning the bi-directional changes of GMV observed in the progression of chronic PTH. Nevertheless, significant CCL2 rise over time and GM volume increase in dPCC were found in patients developing the CPTH, suggesting that the consistently observed associations of inflammation on PTH were at least partially attributable to their effect on GMV alternations. Taken together, the current study highlighted the role of the circulating CCL2 level in the pathogenesis and progression of PTH [48–50], and emphasized on the neuroinflammatory mechanism in morphological alternations associated with pain modulation in cognitive domain.

## Conclusions

In the current study, we demonstrated that GMV alteration can mediate the relationship between the inflammation and pain dysfunction following early mTBI, and such regional change was primarily located within key DMN region (dPCC). Our finding also held the possibility that both the significant upregulation of CCL2 level and persistent GMV increase (dACC and dPCC) only in the patients with CPTH and can be served as potential predictors and targets of response for therapeutic method.

## Supplementary Information

**Additional file 1.**

## Data Availability

The datasets generated during and/or analysed during the current study are available from the corresponding author on reasonable request.

## Abbreviations

dACC: Dorsal anterior cingulate cortex
dPCC: Dorsal posterior cingulate cortex
VLPFC: Ventrolateral prefrontal cortex
OFC: Orbitofrontal cortex
mTBI + APTH: Mild traumatic brain injury and acute post-traumatic headache
CCL2: C-C motif chemokine ligand 2
HIT: Short form headache impact test
mTBI – APTH: Mild traumatic brain injury without acute post-traumatic headache
HCs: Healthy controls
mTBI + CPTH: Mild traumatic brain injury and chronic post-traumatic headache
mTBI – CPTH: Mild traumatic brain injury without chronic post-traumatic headache

## References

[1] Taylor CA, Bell JM, Breiding MJ, Xu L (2017). Traumatic Brain Injury-Related Emergency Department Visits, Hospitalizations, and Deaths - United States, 2007 and 2013. MMWR Surveil Summ.

[2] Jiang JY, Gao GY, Feng JF, Mao Q, Chen LG, Yang XF (2019). Traumatic brain injury in China. Lancet Neurol.

[3] Styrke J, Stalnacke BM, Sojka P, Bjornstig U (2007). Traumatic brain injuries in a well-defined population: epidemiological aspects and severity. J Neurotrauma.

[4] Beetar JT, Guilmette TJ, Sparadeo FR (1996). Sleep and pain complaints in symptomatic traumatic brain injury and neurologic populations. Arch Phys Med Rehabil.

[5] Nordhaug LH, Hagen K, Vik A, Stovner LJ, Follestad T, Pedersen T (2018). Headache following head injury: a population-based longitudinal cohort study (HUNT). J Headache Pain.

[6] Nampiaparampil DE (2008). Prevalence of chronic pain after traumatic brain injury: a systematic review. JAMA..

[7] Johnson VE, Stewart JE, Begbie FD, Trojanowski JQ, Smith DH, Stewart W (2013). Inflammation and white matter degeneration persist for years after a single traumatic brain injury. Brain..

[8] Simon DW, McGeachy MJ, Bayir H, Clark RS, Loane DJ, Kochanek PM (2017). The far-reaching scope of neuroinflammation after traumatic brain injury. Nat Rev Neurol.

[9] Clausen F, Marklund N, Hillered L (2019). Acute inflammatory biomarker responses to diffuse traumatic brain injury in the rat monitored by a novel microdialysis technique. J Neurotrauma.

[10] Mohamadpour M, Whitney K, Bergold PJ (2019). The importance of therapeutic time window in the treatment of traumatic brain injury. Front Neurosci.

[11] Rowe RK, Ellis GI, Harrison JL, Bachstetter AD, Corder GF, Van Eldik LJ (2016). Diffuse traumatic brain injury induces prolonged immune dysregulation and potentiates hyperalgesia following a peripheral immune challenge. Mol Pain.

[12] Obermann M, Nebel K, Schumann C, Holle D, Gizewski ER, Maschke M (2009). Gray matter changes related to chronic posttraumatic headache. Neurology..

[13] Chong CD, Berisha V, Chiang CC, Ross K, Schwedt TJ (2018). Less cortical thickness in patients with persistent post-traumatic headache compared with healthy controls: an MRI study. Headache..

[14] Schrepf A, Kaplan CM, Ichesco E, Larkin T, Harte SE, Harris RE, Murray AD (2018). A multi-modal MRI study of the central response to inflammation in rheumatoid arthritis. Nat Commun.

[15] Miyazawa Y, Takahashi Y, Watabe AM, Kato F (2018). Predominant synaptic potentiation and activation in the right central amygdala are independent of bilateral parabrachial activation in the hemilateral trigeminal inflammatory pain model of rats. Mol Pain.

[16] Witcher KG, Bray CE, Dziabis JE, McKim DB, Benner BN, Rowe RK (2018). Traumatic brain injury-induced neuronal damage in the somatosensory cortex causes formation of rod-shaped microglia that promote astrogliosis and persistent neuroinflammation. Glia.

[17] Smith C, Gentleman SM, Leclercq PD, Murray LS, Griffin WS, Graham DI, Nicoll JA (2013). The neuroinflammatory response in humans after traumatic brain injury. Neuropathol Appl Neurobiol.

[18] Capuron L, Miller AH (2011). Immune system to brain signaling: neuropsychopharmacological implications. Pharmacol Ther.

[19] Sankowski R, Mader S, Valdes-Ferrer SI (2015). Systemic inflammation and the brain: novel roles of genetic, molecular, and environmental cues as drivers of neurodegeneration. Front Cell Neurosci.

[20] Niu X, Bai L (2019). Disruption of periaqueductal grey-default mode network functional connectivity predicts persistent post-traumatic headache in mild traumatic brain injury. J Neurol Neurosurg Psychiatry.

[21] Holm L, Cassidy JD, Carroll LJ, Borg J (2005). Summary of the WHO collaborating Centre for Neurotrauma Task Force on mild traumatic brain injury. J Rehabil Med.

[22] Headache Classification Committee of the International Headache Society (IHS) (2013). The International Classification of Headache Disorders, 3rd edition (beta version). Cephalalgia.

[23] Rathbone AT, Tharmaradinam S, Jiang S, Rathbone MP, Kumbhare DA (2015). A review of the neuro- and systemic inflammatory responses in post concussion symptoms: introduction of the “post-inflammatory brain syndrome” PIBS. Brain Behav Immun.

[24] Miotla Zarebska J, Chanalaris A, Driscoll C, Burleigh A, Miller RE, Malfait AM (2017). CCL2 and CCR2 regulate pain-related behaviour and early gene expression in post-traumatic murine osteoarthritis but contribute little to chondropathy. Osteoarthr Cartil.

[25] Varndell W, Fry M, Elliott D (2017). A systematic review of observational pain assessment instruments for use with nonverbal intubated critically ill adult patients in the emergency department: an assessment of their suitability and psychometric properties. J Clin Nurs.

[26] Kwong WJ, Pathak DS (2007). Validation of the eleven-point pain scale in the measurement of migraine headache pain. Cephalalgia.

[27] Flaherty SA (1996). Pain measurement tools for clinical practice and research. AANA J.

[28] Shin HE, Park JW, Kim YI, Lee KS (2008). Headache Impact Test-6 (HIT-6) scores for migraine patients: Their relation to disability as measured from a headache diary. J Clin Neurol.

[29] Nachit-Ouinekh F, Dartigues JF, Henry P, Becg JP, Chastan G, Lemaire N, El Hasnaoui A (2005). Use of the headache impact test (HIT-6) in general practice: relationship with quality of life and severity. Eur J Neurol.

[30] King NS, Crawford S, Wenden FJ, Moss NE, Wade DT (1995). The Rivermead post concussion symptoms questionnaire: a measure of symptoms commonly experienced after head injury and its reliability. J Neurol.

[31] Martucci KT, Mackey SC (2018). Neuroimaging of pain: human evidence and clinical relevance of central nervous system processes and modulation. Anesthesiology..

[32] Hayes A (2013). Introduction to mediation, moderation, and conditional process analysis. J Educ Meas.

[33] Messina R, Rocca MA, Colombo B, Pagani E, Falini A, Goadsby PJ, Filippi M (2018). Gray matter volume modifications in migraine: a cross-sectional and longitudinal study. Neurology..

[34] Teutsch S, Herken W, Bingel U, Schoell E, May A (2008). Changes in brain gray matter due to repetitive painful stimulation. NeuroImage..

[35] Burrowes SAB, Rhodes CS, Meeker TJ, Greenspan JD, Gullapalli RP, Seminowicz DA (2019) Decreased grey matter volume in mTBI patients with post-traumatic headache compared to headache-free mTBI patients and healthy controls: a longitudinal MRI study. Brain Imaging Behav (2019). 10.1007/s11682-019-00095-7 10.1007/s11682-019-00095-7 PMC7202376 30980274

[36] Strigo IA, Spadoni AD, Lohr J, Simmons AN (2014). Too hard to control: compromised pain anticipation and modulation in mild traumatic brain injury. Transl Psychiatry.

[37] Lavigne G, Khoury S, Chauny JM, Desautels A (2015). Pain and sleep in post-concussion/mild traumatic brain injury. Pain..

[38] Alshelh Z, Marciszewski KK, Akhter R, Di Pietro F, Mills EP, Vickers ER (2018). Disruption of default mode network dynamics in acute and chronic pain states. NeuroImage Clinical.

[39] Baliki MN, Geha PY, Apkarian AV, Chialvo DR (2008). Beyond feeling: chronic pain hurts the brain, disrupting the default-mode network dynamics. J Neurosci.

[40] Leech R, Sharp DJ (2014). The role of the posterior cingulate cortex in cognition and disease. Brain.

[41] Zhou Y, Milham MP, Lui YW, Miles L, Reaume J, Sodickson DK (2012). Default-mode network disruption in mild traumatic brain injury. Radiology..

[42] Truettner JS, Bramlett HM, Dietrich WD (2018). Hyperthermia and mild traumatic brain injury: effects on inflammation and the cerebral vasculature. J Neurotrauma.

[43] Febinger HY, Thomasy HE, Pavlova MN, Ringgold KM, Barf PR, George AM (2015). Time-dependent effects of CX3CR1 in a mouse model of mild traumatic brain injury. J Neuroinflammation.

[44] Raghu H, Lepus CM, Wang Q, Wong HH, Lingampalli N, Oliviero F (2017). CCL2/CCR2, but not CCL5/CCR5, mediates monocyte recruitment, inflammation and cartilage destruction in osteoarthritis. Ann Rheum Dis.

[45] Shetty AK, Mishra V, Kodali M, Hattiangady B (2014). Blood brain barrier dysfunction and delayed neurological deficits in mild traumatic brain injury induced by blast shock waves. Front Cell Neurosci.

[46] Liberto CM, Albrecht PJ, Herx LM, Yong VW, Levison SW (2004). Pro-regenerative properties of cytokine-activated astrocytes. J Neurochem.

[47] Li T, Chen X, Zhang C, Zhang Y, Yao W (2019). An update on reactive astrocytes in chronic pain. J Neuroinflammation.

[48] Zhu X, Cao S, Zhu MD, Liu JQ, Chen JJ, Gao YJ (2014). Contribution of chemokine CCL2/CCR2 signaling in the dorsal root ganglion and spinal cord to the maintenance of neuropathic pain in a rat model of lumbar disc herniation. J Pain.

[49] Illias AM, Gist AC, Zhang H, Kosturakis AK, Dougherty PM (2018). Chemokine CCL2 and its receptor CCR2 in the dorsal root ganglion contribute to oxaliplatin-induced mechanical hypersensitivity. Pain..

[50] Miller RE, Tran PB, Das R, Ghoreishi-Haack N, Ren D, Miller RJ, Malfait AM (2012). CCR2 chemokine receptor signaling mediates pain in experimental osteoarthritis. Proc Natl Acad Sci U S A.

