# Supplementary material for: Mild traumatic brain injury is associated with effect of inflammation on structural changes of default mode network in those developing chronic pain
Source: J Headache Pain. 2020 Nov 23;21(1):135. doi: 10.1186/s10194-020-01201-7 (PMC7684719; doi:10.1186/s10194-020-01201-7)
Supplement: Supplementary file 1 — Additional file 1. [file 10194_2020_1201_MOESM1_ESM.docx]

Supplementary file

**Methods**

***Participants***

Inclusion criteria for all mild TBI patient were based on the World Health Organization’s Collaborating Centre for Neurotrauma Task Force[^1^](#_ENREF_1): i) Glasgow Coma Scale (GCS) score of 13-15 on presentation to the emergency department (ED), ii) one or more/any of the following: loss of consciousness (LOC) for less than 30 minutes, posttraumatic amnesia (PTA) for 24 or less hours, and/or other transient neurological abnormalities such as focal signs, seizure, and intracranial lesion not requiring surgery, iii) within one week after onset of a mild TBI (concussion), iv) were aged 18 years or older. Mild TBI patients were excluded:1) history of a previous brain injury, preexisting headache, neurological disease, long-standing psychiatric condition, or concurrent substance or alcohol abuse, 2) structural abnormality on conventional neuroimaging (CT and MRI), 3) intubation and/or presence of a skull fracture and administration of sedatives, 4) the manifestation of mild TBI due to medications by other injuries (e.g., systemic injuries, facial injuries, or spinal cord injury), 5) other problems (e.g., psychological trauma, language barrier, or coexisting medical conditions), 6) caused by penetrating craniocerebral injury.

The acute posttraumatic headache (APTH) was established according to the Second Edition of the International Classification of Headache Disorders (ICH-D-3)[^2^](#_ENREF_2):

A. Headache, no typical characteristics known

B. Head trauma that includes the following: 1) either no loss of consciousness or loss of consciousness of <30 min duration, 2) Glasgow Coma Scale (GCS) ≥ 13, 3) symptoms and/or signs of concussion as mentioned in the above diagnostic criteria of mTBI

C. Headache occurs within seven days after head trauma

***Image Acquisition***

The MRI scans were acquired on a 3T MRI scanner (GE750) with a 32-channel phase array head coil. A custom-built head holder was used to prevent head movements. The MRI protocol involved the high-resolution structural image by using

T1-weighted 3D BRAVO sequence with the following parameters: echo time (TE) = 3.17 ms, repetition time (TR) = 8.15 ms, flip angle = 9°, slice thickness = 1 mm, field of view (FOV) = 256 mm × 256 mm, matrix size = 256 × 256, acquisition time = 4:30 minutes and resting functional images were obtained by using a single-shot, gradient-recalled echo planar imaging (EPI) sequence with a total of 180 volumes of 54 slices covering the whole brain (TR = 2500 ms, TE = 30 ms, slice thickness = 3 mm, flip angle = 90°, FOV = 216 mm × 216 mm, matrix size = 64 × 64, voxel size = 3 mm × 3 mm × 3 mm, acquisition time = 7:30 minutes). During the resting state fMRI scan, participants were instructed to rest with their eyes closed, not to think about anything in particular, and not to fall asleep. The presence of focal lesions and cerebral microbleeds were independently determined by two experienced clinical neuroradiologists (with 9 and 10 years’ experience) who reviewed multiple modalities of neuroimaging data (T1-flair, T2-flair, T2, susceptibility weighted imaging (SWI)) for all subjects in random sequence. Any disagreement between these two observers was resolved by consensus. None of patients were with visible contusion lesions using conventional neuroimaging techniques or exhibited cerebral micro-bleeds on SWI.

1. Holm L, Cassidy JD, Carroll LJ, et al. Summary of the WHO Collaborating Centre for Neurotrauma

Task Force on Mild Traumatic Brain Injury. *Journal of rehabilitation medicine* 2005;37(3):137-41. doi:

10.1080/16501970510027321 [published Online First: 2005/07/26]

2. Headache Classification Committee of the International Headache Society (IHS) The International

Classification of Headache Disorders, 3rd edition. *Cephalalgia : an international journal of headache*

2018;38(1):1-211. doi: 10.1177/0333102417738202 [published Online First: 2018/01/26]
